# Zn-Quer Nanozymes Reprogram the Malignant Phenotypic Transformation of Gastric Cancer Cells via Cascade Reactive Oxygen Species Coordination

**DOI:** 10.1021/acsami.5c17346

**Published:** 2026-01-08

**Authors:** Heng Jiang, Jiahao Wang, Siyu Gui, Sensen Niu, Guangzheng Lin, Hui Yuan, Yongqi Wu, Chuhan Zhou, Jingjing Tang, Qiao Mei, Lianbang Zhou

**Affiliations:** † Department of General Surgery, The Second Affiliated Hospital of Anhui Medical University, Hefei 230601, China; ‡ Department of Ophthalmology, Shanghai General Hospital, Shanghai Jiao Tong University School of Medicine, Shanghai 200025, China; § Department of Gastroenterology, The First Affiliated Hospitalof Anhui Medical University, Hefei 230022, China; ∥ Department of Urology, The Second Affiliated Hospitalof Anhui Medical University, Hefei 230601, China

**Keywords:** gastric cancer, nanocoordination polymer, quercetin, nanozymes, nanomedicine, reactive oxygen species

## Abstract

Gastric cancer (GC) remains one of the leading causes of cancer-related mortality worldwide, presenting significant therapeutic challenges due to its late-stage diagnosis and high metastatic potential. Imbalance in reactive oxygen species (ROS) homeostasis is crucial for the onset, development, and malignant transformation of GC. To address the demand for innovative therapeutic approaches, this study introduces a novel nanocoordination polymer, Zn-Quer nanozymes (NZs), synthesized from quercetin (Quer) and zinc ions. These nanoparticles utilize quercetin’s anti-inflammatory and antioxidant properties, improving its bioavailability and therapeutic effectiveness via a nanoparticle delivery system. Zn-Quer NZs were synthesized via coordination chemistry and characterized by using TEM, XRD, and FTIR to confirm their structural integrity and composition. In vitro studies on GC cell lines and in vivo xenograft model experiments demonstrate that Zn-Quer NZs effectively inhibit cell proliferation, induce apoptosis, and reprogram malignant phenotypes such as epithelial-mesenchymal transition (EMT) and angiogenesis, as indicated by changes in the expression of related markers. Moreover, Zn-Quer NZs significantly reduce the overall intracellular ROS levels in epithelial cells under oxidative stress by modulating multiple steps of ROS generation. These findings underscore Zn-Quer NZs as a promising therapeutic strategy for gastric cancer, capable of rebalancing ROS and effectively reversing malignant phenotypic transformation.

## Graphical abstract

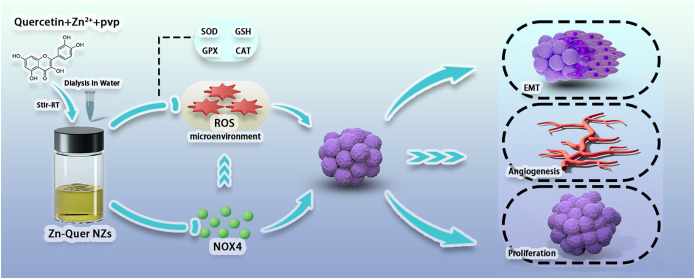

## 1 Introduction

Gastric cancer (GC) remains a formidable global health burden, ranking among the leading causes of cancer-related morbidity and mortality.^1^ Despite advances in surgical interventions and chemotherapeutic regimens, the long-term prognosis for patients with GC remains poor due to late diagnosis, rapid progression, and high rates of metastasis.^2^ A key challenge in gastric cancer (GC) is the imbalance of oxidative stress, largely due to excessive reactive oxygen species (ROS) in the tumor microenvironment.^3^ Excessive ROS generation contributes to DNA damage, inflammation, and aberrant signaling pathways, all of which accelerate tumor growth and dissemination.^4,5^ Intratumoral microenvironmental intervention strategies aimed at multitargeting and flexible coordination of ROS levels have received widespread attention as potential therapeutic avenues to reduce the progression of the GC disease course as well as the differentiation of malignant phenotypes, but relevant protocols are still very limited.^6–9^

Quercetin (Quer), a natural flavonoid prevalent in plants, has garnered significant attention for its antioxidant, anti-inflammatory, and anticancer properties. Quercetin has shown good effects in enhancing the gastric mucosal barrier, reducing inflammation, and inhibiting gastric acid secretion in clinical setting.^10^ It was found that tumor cells with high levels of ROS were more sensitive to the perturbation of cell activity caused by quercetin.^11^ However, the great potential of quercetin in the treatment of cancer is limited by its low bioavailability and poor pharmacokinetics. Its poor water solubility and high polarity lead to its low membrane permeability and oral bioavailability (5.3%), the half-life in vivo is only 11–28 h, and it is more toxic to cells when administered at high concentrations.^12,13^ Therefore, how to deliver sufficient quantities of quercetin to the tumor site without causing off-target toxicity and to leverage its excellent antioxidant pharmacological activity and thus achieve multitargeted control of the pathological microenvironment is a key obstacle to translating its promising bioactivity into effective GC therapies.

Nanotechnology-based formulations offer compelling solutions to the aforementioned obstacles. Nanoparticles offer significant benefits such as the enhanced permeability and retention (EPR) effect, which increases tumor accumulation of therapeutic agents, along with superior loading capacity, prolonged circulation times, and customizable release profiles.^14^ These attributes are essential for ensuring that bioactive compounds, such as quercetin, are delivered to tumor sites at therapeutically effective concentrations, minimizing off-target effects. Among various nanoscale delivery systems, metal–organic frameworks (MOFs) and nanocoordination polymers stand out as exceptionally versatile platforms. Their intrinsically high porosity, tunable pore dimensions, and modifiable chemical compositions allow for efficient drug encapsulation, sustained and stimuli-responsive release, and enhanced pharmacokinetics. Furthermore, the incorporation of metal ions not only stabilizes the framework but also imparts unique functionalities such as catalytic activity, redox reactivity, or ROS modulation, thereby broadening their applicability in cancer theranostics.^15^ Zinc (Zn^2+^) plays a pivotal role in a wide array of enzymatic processes, serving as a catalytic or structural cofactor that supports proper enzyme function. Its versatile coordination chemistry enables the assembly of robust frameworks with precise geometries, which are essential for maintaining the integrity and functionality of nanodelivery platforms under physiological conditions.^16^

In this context, the fabrication of Zn-Quer nanocoordination polymers (NZs) represents a promising approach to overcome quercetin’s delivery challenges and harness its therapeutic potential against GC. Several key aspects of Zn-Quer NZs can directly address the multifaceted challenges in GC treatment. First, ROS modulation constitutes a core mechanism: quercetin’s intrinsic antioxidant capacity attenuates the proinflammatory tumor microenvironment, while zinc coordination enhances both its physicochemical stability and bioactivity. Second, the nanoscale architecture facilitates EPR effects, promoting efficient tumor cell internalization and reducing off-target systemic exposure. Third, beyond their direct cytotoxicity, these nanoformulations suppress pro-angiogenic signaling pathways that drive pathological neovascularization, thereby limiting tumor vascularization and nutrient access. Finally, through the disruption of molecular pathways governing proliferation and invasiveness, Zn-Quer NZs exhibit the potential to curtail metastatic progression and improve therapeutic outcomes.

In view of the above background, the aim of this study was to establish a set of stable and novel nanoparticle synthesis routes by coordinating Quer with Zn ions using MOF nanosynthesis technology, characterize them by TEM, XRD, and FTIR in order to confirm the structural integrity and composition of Zn-Quer NZs, detect their inhibitory effects on the proliferation and apoptosis levels of cancer cells in in vitro gastric cancer cell line experiments and in vivo xenograft tumor models, and further test its ability to reprogram malignant phenotypes such as EMT and angiogenesis, as well as specific pathways. The study will address the key formulation challenges associated with quercetin through the design of nanocoordination polymers, introduce a platform that can regulate ROS levels, inhibit angiogenesis, and inhibit tumor progression through multiple molecular pathways, and establish whether Zn-Quer NZs can be an effective and innovative therapy for the treatment of GC.

## 2 Materials and Methods

### 2.1 Materials and Reagents

Quercetin (≥98%; Sigma-Aldrich, cat. Q4951) and zinc chloride hexahydrate (ZnCl_2_·6H_2_O; Sigma-Aldrich, cat. 96468) were purchased from Sigma-Aldrich. Polyvinylpyrrolidone (PVP, MW ≈ 40,000; Aladdin, cat. P112746) was obtained from Aladdin.DMEM (Gibco, cat. 11965–092), RPMI-1640 (Gibco, cat. 11875–093), fetal bovine serum (FBS; Gibco, cat. 10099141C), penicillin–streptomycin (Gibco, cat. 15140122), and trypsin-EDTA (Gibco, cat. 25200056) were all purchased from Thermo Fisher Scientific. The Cell Counting Kit-8 (CCK-8) was purchased from Dojindo Molecular Technologies (cat. CK04).

AGS human gastric adenocarcinoma cells were purchased from the American Type Culture Collection (ATCC, Manassas, VA, USA; cat. CRL-1739). MKN-45 gastric carcinoma cells (Procell, Wuhan, China; cat. CL-0292) and GES-1 gastric epithelial cells (Procell; cat. CL-0563) were obtained from Procell Life Science & Technology Co., Ltd. All cell lines were authenticated by STR profiling and routinely tested negative for mycoplasma contamination.

Primary antibodies included anti-NOX4 (Cell Signaling Technology, #14347), anti-VEGF (CST, #2463), anti-HIF-1α (CST, #14179), anti-E-cadherin (CST, #14472), anti-N-cadherin (CST, #13116), anti-Vimentin (CST, #5741), anti-BCL-2 (CST, #4223), anti-BAX (CST, #2772), anticleaved Caspase3 (CST, #9664), and β-actin (CST, #4970). Secondary antibodies included Alexa Fluor 488 goat antirabbit IgG (H+L) (Thermo Fisher Scientific, cat. A-11008) and Alexa Fluor 488 goat antimouse IgG (H+L) (Thermo Fisher Scientific, cat. A-11001).

### 2.2 Synthesis of Zn-Quer NZs

Dissolve 20 mg of ZnCl_2_·6H2O in 1 mL of methanol and add it to a solution of 66 mg of polyvinylpyrrolidone (PVP) in 5 mL of methanol. Stir the mixture rapidly for 5 min to obtain solution A. Solution B was prepared by adding 1 mL of methanol dropwise to 10 mg of quercetin, followed by 5 min of ultrasonic mixing. Gradually add solution A to solution B, stirring continuously for 3 h to form methanol solution C. Dialyze solution C in a dialysis strip with sufficient pure water overnight (12–16 h) to yield aqueous solution D. Measure the metal ion content using single-channel scanning optoelectronic direct reading spectrometry (ICP-AES) to determine the concentration of Zn-Quer NZs. The concentration was fixed to the same concentration for further characterization and screening.

### 2.3 Characterization

TEM images were captured using TEM (FEI Tecnai F20, 200 kV). Absorption spectra in the UV–vis-NIR range were measured by using a PerkinElmer spectrophotometer. The dynamic light scattering (DLS) of CPN was analyzed by using a Malvern Nano ZS 90 instrument. XPS measurements were determined using the metal content obtained through inductively coupled plasma atomic emission spectrometry (ICP-OES). FTIR spectra were obtained using a Bruker Vertex 70 spectrometer, covering the range of 400 to 4000 cm^–1^.

### 2.4 Free Radical Scavenging Experiment

#### 2.4.1 ABTS Assay

ABTS radicals were activated by incubating a 7 mM ABTS solution with 2.45 mM potassium persulfate overnight. Zn-Quer NZs at concentrations of 0, 2, 4, 8, and 16 μg/mL were combined with ABTS radical solution and incubated for 10 min. The absorption of ABTS radicals was measured at a wavelength of 734 nm. The scavenging efficiency of the ABTS radical was calculated according to the following formula. The ABTS clearance efficiency percentage is calculated using the formula ((*A*_ABTS_ – *A*_Sample_)/*A*_ABTS_) × 100%. *A*_ABTS_ denotes the untreated absorbance of the ABTS. *A*_Sample_ denotes the absorbance of ABTS following the addition of the Zn-Quer NZs.

#### 2.4.2 DPPH Assay

A solution was prepared by dissolving 1 mg of DPPH in 24 mL of ethanol, followed by sonication for 5 min. Zn-Quer NZs at concentrations of 0, 2, 4, 8, and 16 μg/mL were combined with a DPPH radical solution and incubated for 30 min. The absorption of DPPH was measured at 519 nm. DPPH removal efficiency is calculated according to the following formula. DPPH clearance efficiency (%) = ((*A*_DPPH_ – *A*_Sample_)/*A*_DPPH_) × 100%. *A*_DPPH_ represents the absorbance of DPPH without an additional treatment. *A*_Sample_ represents the absorbance of DPPH after addition of Zn-Quer NZs.

#### 2.4.3 MB Assay

Zn-Quer NZs at concentrations of 0, 2, 4, 8, and 16 μg/mL were combined with the MB solution, followed by the addition of a Fenton reaction solution containing H_2_O_2_ and Zn^2+^. After a 30 min incubation, absorbance was measured at 665 nm. The efficiency of MB radical scavenging was determined using the specified formula. MB clearance efficiency (%) is calculated as (*A*_Sample_/*A*_MB_) × 100%. *A*_MB_ indicates the absorbance of methyl bromide without any further treatment. *A*_Sample_ denotes the MB absorbance after Zn-Quer NZs addition.

#### 2.4.4 PTIO Assay

Dissolve a certain concentration (such as 1 mm) of PTIO in PBS, prepare Zn-Quer NZs into different concentrations of aqueous solutions (0, 2, 4, 8, 16 μg/mL), mix with different concentrations of PTIO solutions, incubate for 30 min after full stirring, and measure the absorbance at 560 nm using UV-vis spectrometer. The PTIO removal efficiency (%) is calculated using the formula ((*A*_PTIO_ – *A*_Sample_)/A_PTIO_) × 100%, where *A*_PTIO_ denotes the absorbance of the PTIO solution without Zn-Quer nanoparticles, and *A*_Sample_ indicates the absorbance of the PTIO solution after the addition of Zn-Quer NZs.

#### 2.4.5 ^•^OH Scavenging Assay

The TEMPO radical exhibited a distinctive peak signal with an intensity ratio of 1:2:2:1 for the hydroxyl radicals (^•^OH). Zn-Quer NZs were introduced to a mixture containing H_2_O_2_, Zn^2+^, and DMPO solutions for 30 s. The characteristic peak signal of ^•^OH was measured by an ESR spectrometer.

#### 2.4.6 ^•^O_2_ Scavenging Assay

TEMPO radicals showed a characteristic peak signal with a singlet oxygen (^•^O2) intensity ratio of 1:1:1. Zn-Quer NZs were combined with TEMPO solution for 1 min, followed by measurement of the characteristic peak signal using an ESR spectrometer.

### 2.5 Enzymomimetic Activity Assay

#### 2.5.1 SOD-like Activity Assay

The SOD-like activity of Zn-Quer NZs was analyzed spectrophotometrically using a SOD detection kit, following the manufacturer’s guidelines. The core mechanism involves the generation of superoxide anion (O_2‑_) through the reaction of xanthine with the xanthine oxidase enzyme system. This O_2‑_ reduces azotetrazole, resulting in the formation of blue formazan, which can be measured at 560 nm. Superoxide dismutase (SOD) scavenges the ligands of O_2‑_, thus inhibiting formazan production. Consequently, a deeper blue color in the reaction solution indicates lower SOD activity, whereas a lighter color signifies higher SOD activity. To assess the O_2‑_ scavenging capability of Zn-Quer NZs, a mixture of WST-8, xanthine, and XOD was prepared in Zn-Quer NZs dispersions at varying concentrations (0, 2, 4, 8, and 16 μg/mL). The absorption variations of water-soluble formazan were monitored spectrophotometrically at 450 nm using a UV–vis-NIR spectrometer.

#### 2.5.2 GSH-like Activity Assay

GSH-like activity assay oxidizes the thiol reagent 5,5′-Dithiobis (2-nitrobenzoic acid) (DTNB) to produce the yellow derivative 5′-thio-2-nitrobenzoic acid (TNB), which can be detected at a wavelength of 412 nm. Various concentrations (0, 2, 4, 8, 16 μg/mL) of Zn-Quer NZS were reacted with DTNB, and the resulting TNB absorption changes were spectrophotometrically monitored at 412 nm using a UV-vis NIR spectrometer with three replicates for each concentration.

#### 2.5.3 GPX-like Activity Assay

GPX-like enzymes facilitate the conversion of hydrogen peroxide or lipid peroxides into water or alcohols using reduced glutathione (GSH), which is simultaneously oxidized to form oxidized glutathione (GSSG). Glutathione reductase reduces GSSG to GSH using NADPH, which is oxidized to NADP+, resulting in a decreased absorbance at 340 nm. The GPX-like activity can be indirectly assessed by using a UV-vis NIR spectrometer to measure the absorption spectrum at 340 nm of the reaction product with varying concentrations (0, 2, 4, 8, and 16 μg/mL) of Zn-Quer NZS.

#### 2.5.4 CAT-like Activity Assay

The CAT-like activity of Zn-Quer NZs was assessed spectrophotometrically using the CAT detection kit as per the manufacturer’s guidelines. H_2_O_2_ exhibits a distinct absorption peak at 240 nm. As CAT decomposes H_2_O_2_, the absorbance at this wavelength diminishes over time, allowing the CAT activity to be determined by the rate of absorbance change. The consumption of H_2_O_2_ with Zn-Quer NZs was quantified by directly measuring the absorption spectrum at 240 nm using a UV-vis NIR spectrophotometer with three samples per group.

### 2.6 Cell Culture and Treatment

Cells were cultured in RPMI 1640 medium supplemented with 10% fetal bovine serum, 1% l-glutamine, and penicillin/streptomycin (all from GIBCO, Waltham, MA, USA) at 37 °C with 5% CO_2_. The culture medium was replaced every 3 days, and cells were passaged upon reaching 70%–90% confluence. During the logarithmic growth phase, varying doses of Zn-Quer NZs or an equivalent volume of cell culture medium (control group) were administered to each group to assess morphological changes and conduct functional tests, identifying the optimal time point for observing drug intervention effects.

### 2.7 Intracellular ROS Detection

Intracellular ROS levels were assessed by using a DCFH-DA oxidation-sensitive fluorescent dye kit. DCFH-DA has no fluorescence and can freely cross the cell membrane. Once it enters the cell, it can be hydrolyzed by cellular lactonase to produce DCFH. DCFH is impermeable to the cell membrane, making it easier for the probe to be loaded into the cell. Intracellular reactive oxygen species levels can be assessed by measuring the fluorescence intensity of DCF, produced through the oxidation of nonfluorescent DCFH by these species. DCFH-DA was diluted with serum-free medium at a ratio of 1:1000, and the final concentration was 10 μmol/L. Cells suspended in diluted DCFH-DA were collected to a concentration of 100–2000/ml and incubated at 37 °C for 20 min. Invert every 3–5 min to ensure complete contact between the probe and the cell. The cells were rinsed three times with serum-free culture medium to completely eliminate any extracellular DCFH-DA. Cells were incubated with DAPI staining solution for 15 min at room temperature, followed by three 10 min washes with PBS containing 0.1% Tween-20. Fluorescence intensity for each well was recorded within 30 min using a Thermo Scientific Varioskan LUX microplate reader, a flow cytometer, and a laser confocal microscope. Fluorescence intensity was measured at each time point or in real time using an excitation wavelength of 488 nm and an emission wavelength of 525 nm, both before and after stimulation. The fluorescence spectrum of DCF closely resembles that of FITC, allowing detection with the FITC parameter settings.

### 2.8 Detection of the Apoptosis Level

GES-1 cells were plated at 2 × 10^5^ cells per well in 6-well plates and incubated overnight. After treatment with the test compounds, cells were harvested and resuspended in a binding buffer. Following the manufacturer’s protocol, the cell suspension was stained using Annexin V-FITC and propidium iodide (PI). Cells were incubated in the dark at room temperature for 15 min with 5 μL each of Annexin V-FITC and PI. Following staining, cells were washed and resuspended in binding buffer. Cells were examined with a fluorescence microscope using excitation/emission wavelengths of 488/520 nm for FITC and 535/617 nm for PI fluorescence. Cell viability was assessed by quantifying early apoptotic (Annexin V+/PI-), late apoptotic or necrotic (Annexin V+/PI+), and viable (Annexin V-/PI-) cells through fluorescence analysis in various random fields.

### 2.9 Cell Viability Assay (CCK-8)

CCK-8 method was used to study the cytotoxicity of Zn-Quer NZs and its effect on cell viability. MKN-45 and AGS cells were seeded into 96-well plates at a concentration of 8 × 10^4^ cells/mL, with a volume of 100 μL per well. Following a 24 h incubation at 37 °C in a humidified environment with 5% CO2 and 95% air, the medium was refreshed with 100 μL of new medium containing varying concentrations (0 to 256 μg/mL) of Zn-Quer NZs. After 24 h of culture, the cells were gently rinsed twice with prewarmed PBS. A total of 100 μL of fresh medium with 10 μL of CCK-8 working solution was added and incubated for 2 h. Absorbance was then measured at 450 nm using a Spectra M2 microplate reader (Molecular Devices, CA, USA) to calculate cell viability. The EC50 of the Zn-Quer NZs was calculated according to the dose effect curve.

### 2.10 Clonogenic Assay

Cells in the logarithmic growth phase were trypsinized with 0.25% trypsin to form a single-cell suspension and then resuspended in 10% fetal bovine serum for subsequent use. The cell suspension was serially diluted and inoculated into culture dishes at an optimal cell density based on the proliferation capacity. Cell densities of 50, 100, and 200 per dish were individually introduced into dishes with 10 mL of prewarmed culture solution at 37 °C and gently rotated to ensure even cell distribution. Zn-Quer NZs was added at varying concentrations and incubated for 2–3 weeks at 37 °C in 5% CO2 with saturated humidity. The culture was terminated once visible clones appeared in the dish. The supernatant was discarded and carefully washed twice with PBS. Add 5 mL of either pure methanol or a 1:3 acetic acid/methanol mixture and fix for 15 min. After removing the fixation solution, apply Giemsa stain for 10–30 min, rinse gently with running water, and air-dry. Invert the plate and place a transparent grid film over it to count the clones visually or use a low-power microscope to count clones with more than 10 cells. Finally, the clonogenic rate was calculated.

### 2.11 Wound-Healing Migration Assay

An artificial gap is created in the fused monolayer cells, prompting edge cells to migrate into the gap to repair the “scratch” or “wound.” Cells at the periphery progressively migrate into the vacant region to repair the scratch or wound. Upon reaching over 95% cell density in each group of monolayer cells in a six-well plate, various concentrations of Zn-Quer NZs were added to the culture medium. The central area of cell growth was then marked with a micropipette tip, and cells were subsequently washed out with PBS. The mobility index (%) is calculated by dividing the area of migrated cells by the area enclosed by the double vertical dashed lines. Relative mobility (%) = number of migrated cells/number of migrated cells in DC group.

### 2.12 Transwell Migration Assay

Add serum-free medium into the 24 well plate according to 200 μL/well, slowly put it into the Transwell chamber (Corning), and no bubbles can be left between the bottom of the chamber and the liquid level of the medium; Resuspend the counted cells in logarithmic growth phase at 30,000 cells/600 μL and mix them gently with a Pasteur tube. Introduce 600 μL of cell suspension into the upper section of each Transwell chamber, apply varying concentrations of Zn-Quer NZs, and incubate at 37 °C with 5% CO_2_ for 12, 24, or 48 h. Carefully remove the chamber using forceps, gently clear the cells from the bottom of the upper chamber with a cotton swab, rinse twice with PBS, and fix with 4% paraformaldehyde for 10 min at room temperature. Rinse again with PBS, stain with 1% crystal violet for 30 min at room temperature, and wash repeatedly with PBS until no visible staining remains outside the lower chamber. Invert and allow to air-dry naturally. Capture images using a standard light microscope.

### 2.13 Tube Formation Assay

To evaluate the angiogenic potential of endothelial cells, a μ-slide angiogenesis plate (ibidi) was utilized, and ABW gold matrix gel (low growth factor) was thawed overnight at 4 °C prior to the experiment. On the test day, each well of the ibidi angiogenic slide received 10 μL of ABW gold matrix gel (low growth factor), which was quickly spread by rotation and incubated at 37 °C for 30 min. Cells at 70%–90% confluence in the logarithmic growth phase were centrifuged at 1200 rpm and resuspended in a specialized medium to achieve a density of 2 × 10^5^ cells/ml. Each well received 50 μL of HUVEC, while the coculture group also included 1/10 cell counting MKN-45 cell suspension and varying concentrations of Zn-Quer NZs, all incubated at 37 °C in a carbon dioxide incubator.

### 2.14 Transplanted Tumor Model

Four-week-old female BALB/c nude mice, each weighing approximately 13 g, were randomly divided into four experimental and four control groups. For both groups, two T75 flasks of cells in the logarithmic growth phase were prepared. The original culture medium was discarded, and the cells were washed twice with PBS. Cells were digested with 1 mL of 0.5% trypsin at 37 °C until they became round, then gently tapped to suspend them. To stop digestion, 3 mL of complete medium was added per flask, and the contents were transferred to a 15 mL centrifuge tube, adjusted to 5 mL, and mixed to form a single-cell suspension. A 10 μL sample was taken for counting and labeling. The suspension was centrifuged at 800 rpm for 5 min at room temperature, and the supernatant was discarded. Based on the cell count, PBS was added to achieve a concentration of approximately 1 × 10^7^ cells/ml. The suspension was mixed thoroughly, wrapped in gauze, and stored on ice. Mice were restrained, and the cell suspension was mixed again before 150 μL was drawn into a 1 mL microinjector. Using the fourth pair of mammary glands in the right lower abdomen as the injection site, 100 μL of the suspension was injected subcutaneously at a 15° angle. After needle withdrawal, the injection site was pressed with a cotton swab to prevent leakage. The control group underwent the same procedure. Postinoculation, the mice were fed routinely. Tumor size was measured with a caliper every 3 days, and volume was calculated using the formula: volume = (tumor length × width^2^)/2. Simultaneously, the body weight of the nude mice was recorded. Animal experiments received ethical approval from the Anhui Medical University Laboratory Animal Ethics Committee (LLSC20200503).

### 2.15 Animal Intervention

The treatment group received a daily oral dose of 25 mg/kg Zn-Quer NZs (200 μL), while the control group was administered an equivalent volume of normal saline and Quercetin solution by gavage. After the largest tumor grew to 1.5 cm in diameter, the tumor was sacrificed with 0.5% sodium pentobarbital plus high concentration carbon dioxide, dissected, photographed, and pictured. The specimen was stored in 4% paraformaldehyde solution, and the corpse was treated harmlessly.

### 2.16 Immunofluorescence

Store mouse tumor tissue in 4% paraformaldehyde and embed it in paraffin. Slice the paraffin block to obtain a tissue sample with a thickness of 3 μm and then deparaffinize, hydrate, and wash the sample with water. Treat with 0.3% Triton X-100 (SunShine Biotechnology Co), then incubate overnight at 4 °C with antibodies against E-Cadherin, N-Cadherin, and Vimentin, followed by a 1 h incubation with corresponding fluorescently labeled secondary antibodies (Invitrogen) at room temperature. The cell nucleus was stained with 4′,6-diamidino-2-phenylindole (DAPI, Beyotime, China) for 10 min, followed by washing and analysis using fluorescence microscopy (Leica DMi8, Leica Biosystems Inc.). The same method is used for the immunofluorescence of cell slides.

### 2.17 Western Blot

Protein samples were resolved using 10% or 15% SDS-PAGE and subsequently transferred to PVDF membranes. The membranes were blocked in TBST (Tris Buffered Saline, 0.1% Tween 20 detergent) buffer containing 5% BSA for 2 h and then incubated with primary antibodies overnight at 4 °C. Subsequently, the membrane underwent two washes with TBST, and protein bands were visualized using enhanced chemiluminescence.

### 2.18 RT-qPCR

Total RNA was extracted using Trizol, followed by complementary DNA synthesis with the RT Kit (TaKaRa, Tokyo, Japan) as per the manufacturer’s instructions. FastStart Universal SYBR Green Master (Rox; Roche, Basel, Switzerland) and PrimeScript were also utilized according to the provided guidelines. Using a CFX96 real-time system and C1000 RT-qPCR, reactions were performed with a thermal cycler (Bio-Rad, Hercules, CA, USA). Primers were synthesized by SANGON Biotechnology (Shanghai, China). Relative mRNA expression fold change was determined using the 2-ΔΔCt comparative threshold cycle method. The target gene expression was standardized using the reference gene β-actin. Each PCR was conducted three times. The primer sequences used for RT-qPCR are provided in Table S1.

### 2.19 Histology (H&E Staining)

Mice organs, prepared as outlined in Section 2.13, were preserved in 10% buffered formalin, paraffin-embedded, and stained with hematoxylin and eosin in 5 μm sections. Histological changes in the sections were examined using a light microscope. Mouse tissues, including the heart, liver, and kidney, were stained using a Ventana Nexes automatic staining machine (Ventana, USA).

### 2.20 Statistical Analysis

Quantitative results are expressed as the mean ± standard deviation. Each average represents the average of all of the analyzed experimental groups. Statistical analyses were compared by independent reviewers. Group differences were statistically assessed using one-way or two-way ANOVA, followed by Tukey’s post hoc test. Results were statistically significant at *p < 0.05, **p < 0.01, ***p < 0.001, and ****p < 0.0001. GraphPad Prism 8.0 software was used for statistical significance analysis.

## 3 Results

### 3.1 Synthesis and Characterization of Zn-Quer NZs

A comprehensive series of characterization techniques was performed to validate the successful synthesis of Zn-Quer NZs. As shown in (Figure 1a), the Zn-Quer NZs displayed a uniform yellow color in solution, indicating the formation of well-defined nanoparticles. Transmission electron microscopy (TEM) revealed discrete spherical particles (Figure 1b), and a Gaussian fit to the measured diameters indicated Zn-Quer NZs size ranged from 1 to 4 nm (Figure 1c). Furthermore, we used dynamic light scattering to detect the nanoscale size (Figure S1A), and the results were consistent with those described above. Considering that the potential of nanomedicines is also a factor influencing drug efficacy, we also measured the potential of Zn-Quer (Figure S1B), where Zn-Quer exhibited a stable −4.88 mV.

**Figure 1:**
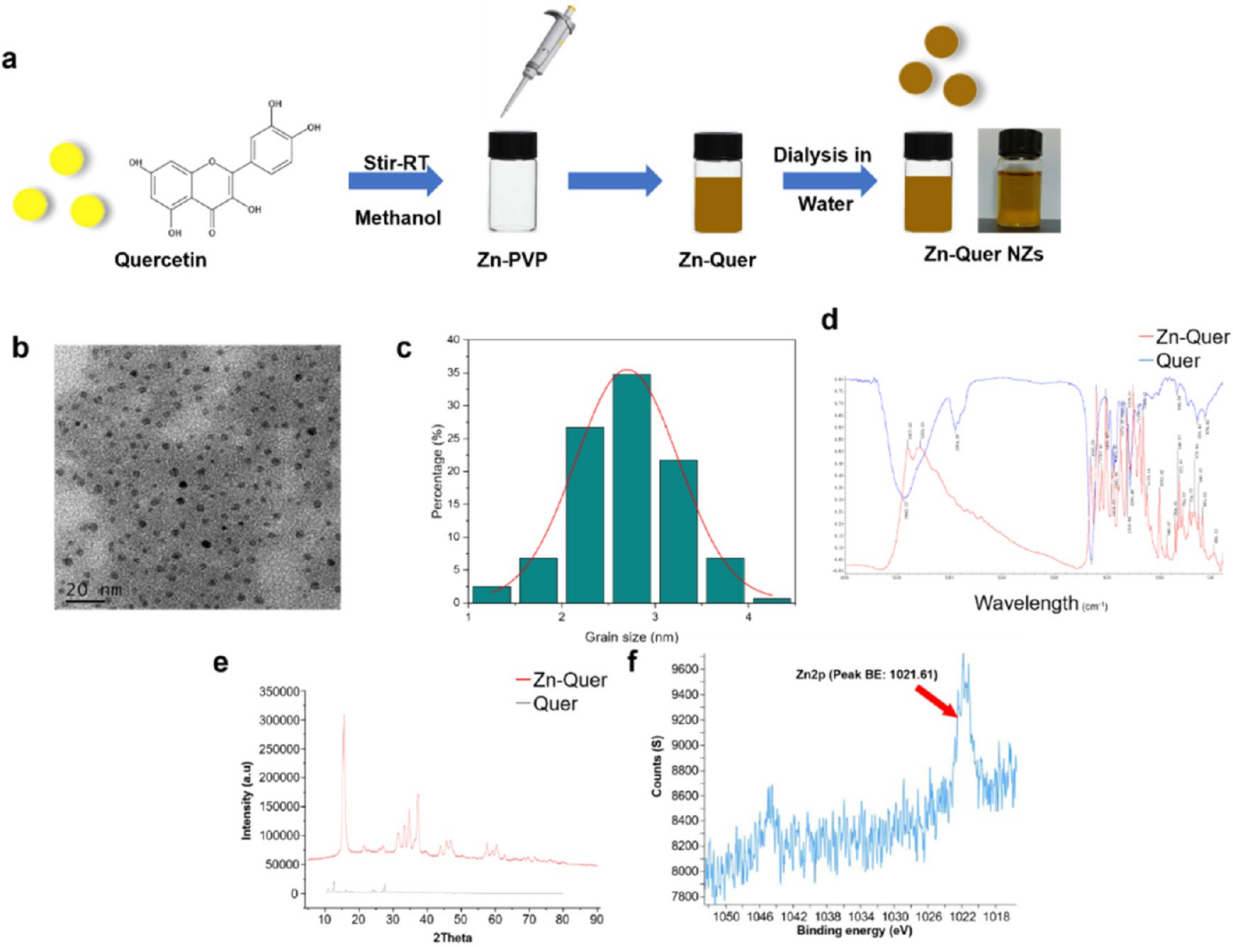
Synthesis and characterization of Zn-Quer NZs. (a) Synthesis procedure and overall appearance of Zn-Quer NZs. (b) TEM images indicating spherical morphology. (c) Diameter distribution based on TEM measurements. (d) FTIR spectra illustrating distinct shifts in quercetin peaks after Zn^2+^ coordination. (e) XRD patterns validating the crystalline nature of Zn-Quer NZs. (f) XPS spectrum showing the Zn 2p peak at 1021.61 eV.

Dynamic light scattering confirmed the hydrodynamic size of the Zn-Quer NZs, aligning with TEM observations. Fourier-transform infrared spectroscopy (FTIR) revealed a reduction in the infrared intensity of the HO-C stretching band within the 1150 to 1200 cm^–1^ range, signifying effective coordination between Zn^2+^ ions and the HO-C groups of quercetin (Figure 1d). The XRD pattern for Zn-Quer NZs demonstrates distinct peaks that indicate the crystalline structure of the nanoparticles. The comparison with pure quercetin suggests successful coordination between zinc ions and quercetin, as evidenced by the differences in peak positions and intensities between Zn-Quer NZs and the original quercetin sample (Figure 1e). The X-ray photoelectron spectroscopy (XPS) spectrum of Zn 2p shows a peak at a binding energy of 1021.61 eV. This peak confirms the presence of zinc ions within the Zn-Quer NZs, indicating the successful incorporation of zinc in the nanoparticle formulation. The specific binding energy peak provides insight into the chemical state of zinc in the nanoparticles, supporting the coordination interaction with quercetin molecules (Figure 1f). Thus, we developed a concise and efficient approach to synthesizing a highly water-soluble nanocoordination compound.

### 3.2 Capacity of Zn-Quer NZs with Multiple Enzyme-Mimetic Properties to Coordinate ROS Levels during the Generation and Degradation Phases

The capacity of Zn-Quer NZs to scavenge reactive oxygen species (ROS) and mimic enzymatic activity was investigated through various assays. A dose-dependent radical-quenching effect was observed for ABTS, DPPH, PTIO, and MB, as depicted in Figure 2a–d, with higher Zn-Quer NZ concentrations delivering significantly enhanced scavenging. Furthermore, ESR confirmed the effective trapping of hydroxyl (^•^OH) and superoxide (O_2_^–^) radicals (Figure 2e–f), and quantitative analyses revealed a substantial scavenging efficiency (Figure 2g–h).

**Figure 2:**
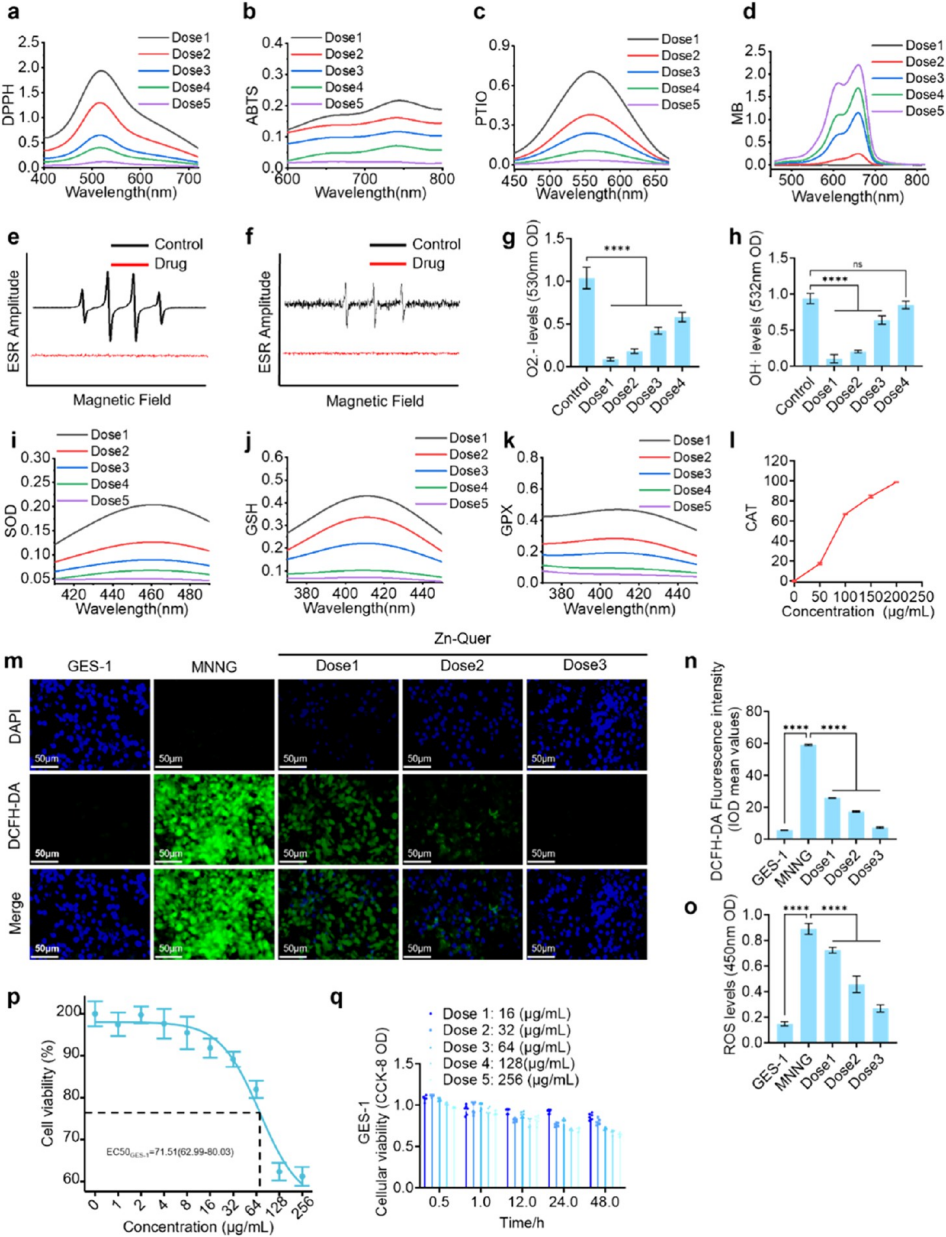
ROS-scavenging ability and enzymomimetic activity of Zn-Quer NZs in vitro. (a–d) Dose-dependent scavenging of ABTS, DPPH, PTIO, and MB radicals (Dose1 = 0 μg/mL, Dose2 = 2 μg/mL, Dose3 = 4 μg/mL, Dose4 = 8 μg/mL, Dose5 = 16 μg/mL; *n* = 6). (e–f) ESR spectra confirming the capture of ·OH and O_2_^–^ radicals­(Zn-Quer = 16 μg/mL, *n* = 6). (g–h) Quantitative analyses of radical scavenging (Dose1 = 16 μg/mL, Dose2 = 8 μg/mL, Dose3 = 4 μg/mL, Dose4 = 2 μg/mL; *n* = 6). (i–l) SOD-, GSH-, GPX-, and CAT-like activities measured in Zn-Quer NZs (Dose1 = 0 μg/mL, Dose2 = 2 μg/mL, Dose3 = 4 μg/mL, Dose4 = 8 μg/mL, Dose5 = 16 μg/mL; *n* = 6). (m–n) Fluorescence images of GES-1 cells and ROS quantification (DCFH-DA) (Dose1 = 4 μg/mL, Dose2 = 8 μg/mL, Dose3 = 16 μg/mL; *n* = 3). (o) Fluorescence zymography illustrating ROS reduction (Dose1 = 4 μg/mL, Dose2 = 8 μg/mL, Dose3 = 16 μg/mL; *n* = 6). (p) CCK-8 assays demonstrate the dose–response curve for GES-1 cell viability after 48-h treatment with varying concentrations of Zn-Quer NZs (*n* = 6). (q) CCK-8 assays showing the cell viability of GES-1 treated with Zn-Quer NZs at different concentrations and times (*n* = 6).

The enzyme-like functionalities of Zn-Quer NZs were characterized by measuring SOD-, GSH-, GPX-, and CAT-like activities, shown in Figure 2i–l, each displaying notable catalytic behaviors. Stimulate GES-1 cells with 200 μmol/L MNNG for 48 h to simulate a chronic inflammatory state in cells, and choose the three largest concentrations of Zn-Quer NZs with cell activity greater than 90%. Intracellular ROS modulation was assessed in GES-1 cells, the fluorescence images in Figure 2m highlight a marked reduction in ROS with increasing Zn-Quer NZ doses, and the quantification (Figure 2n) aligns with this trend. Fluorescence zymography (Figure 2o) further corroborated these antioxidant properties.

The CCK-8 assay indicated low cytotoxicity with cell viability around 70% at concentrations up to 71.51 μg/mL (EC50) (Figure 2p). Over a 48-h period, GES-1 cell viability at nontoxic concentrations showed little variation (Figure 2q). Collectively, these results demonstrate that Zn-Quer NZs possess potent ROS-scavenging and enzyme-mimetic attributes while maintaining favorable biocompatibility.

### 3.3 Zn-Quer NZs Inhibit the Pathological Angiogenic Properties of GCs

The antiangiogenic potential of Zn-Quer NZs was evaluated by monitoring vascular formation and assessing the expression of key angiogenesis-related markers. Figure 3a illustrates a significant reduction in neovascularization with increasing Zn-Quer NZs concentrations, resulting in fewer and more fragmented blood vessel networks compared to those of untreated controls.

**Figure 3:**
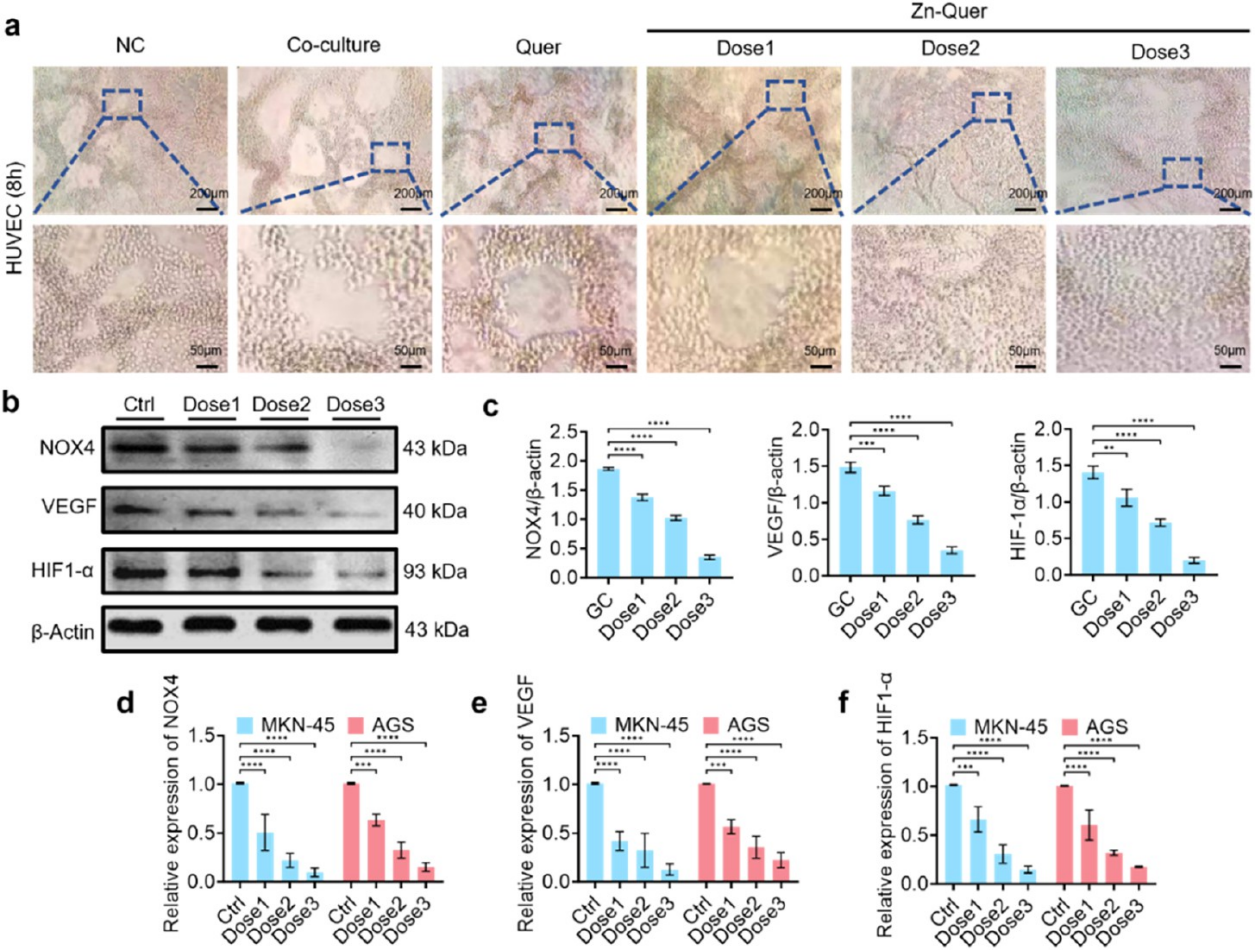
Zn-Quer NZs inhibit angiogenesis in GC. (a) Representative images demonstrating reduced blood vessel formation (Dose1 = 4 μg/mL, Dose2 = 8 μg/mL, Dose3 = 16 μg/mL). (b) Western blot detection of NOX4, VEGF, and HIF1-α. (c) Quantification of protein levels in (b) (*n* = 3). (d, f) RT-qPCR analyses of NOX4, VEGF, and HIF1-α mRNA expression (*n* = 3).

On the molecular level, protein expression of NOX4, VEGF, and HIF1-α was detected via Western blot (Figure 3b). Densitometric analyses (Figure 3c) showed marked decreases in these proteins, whereas RT-qPCR confirmed the dose-dependent suppression of their corresponding mRNAs (Figure 3d–f). These data collectively indicate that Zn-Quer NZs impede angiogenic pathways at both transcriptional and translational levels.

### 3.4 Zn-Quer NZs Reprogram GC Cells Proliferative Viability as Well as Malignant EMT

The study investigated the suppression of gastric cancer cell proliferation and the epithelial-mesenchymal transition (EMT) by Zn-Quer NZs in MKN-45 and AGS cells. As presented in Figure 4a–b, cell viability decreased markedly with higher Zn-Quer NZ doses and longer incubation periods. A significant reduction in proliferation at 48 h was observed in the dose–response curves (Figure 4c). To verify that Zn-Quer is superior to single-drug therapy, we used compuSyn software to analyze CCK-8 cell data (Figure S2A,B). The results showed that Zn-Query has an excellent combination therapy efficacy. Clonogenic assays (Figure 4d) supported these findings, and quantification of colony counts (Figure 4f) demonstrated statistically significant differences from controls.

**Figure 4:**
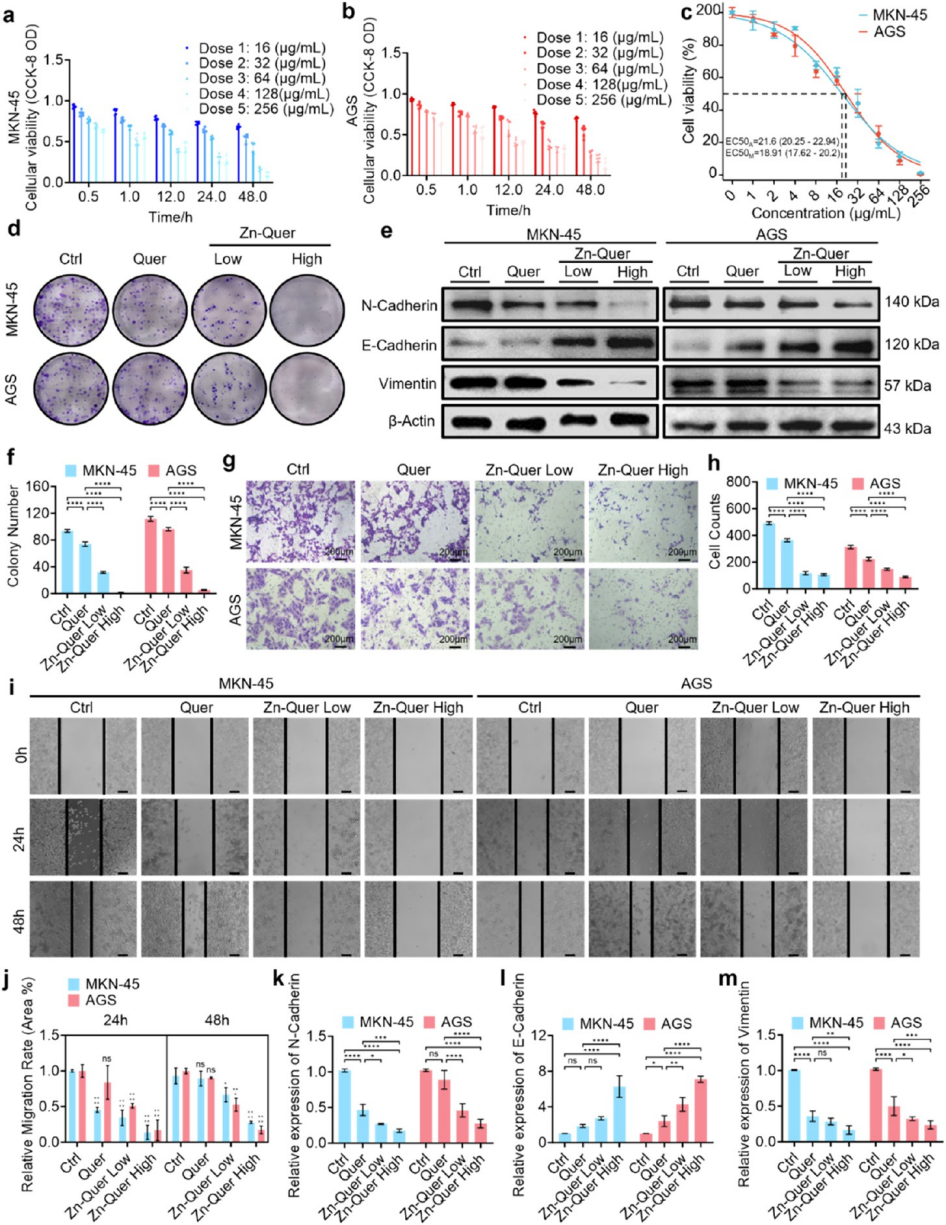
Zn-Quer NZs suppress the proliferation and epithelial-mesenchymal transition of gastric cancer cells. (a–b) CCK-8 assays of MKN-45 and AGS cells under various doses and times (*n* = 6). (c) Dose–response viability curves at 48 h (*n* = 6). (d) Plate colony formation assays reflecting lowered proliferative capability (Zn-Quer Low = 4 μg/mL, Zn-Quer High = 16 μg/mL). (e) Western blot of N-Cadherin, E-Cadherin, and Vimentin. (f) Quantification of colony numbers from (d) (*n* = 3). (g) Transwell images showing reduced invasion after Zn-Quer NZs treatment (Zn-Quer Low = 4 μg/mL, Zn-Quer High = 16 μg/mL). (h) Densitometric analysis of Transwell and Western blot data (*n* = 3). (i) Wound-healing assays demonstrating impaired cell migration (Zn-Quer Low = 4 μg/mL, Zn-Quer High = 16 μg/mL; scale bar: 20 nm). (j) Relative migration rates derived from (i) (*n* = 3). (k,m) RT-qPCR quantification of N-Cadherin, E-Cadherin, and Vimentin mRNA (*n* = 3).

Regarding EMT markers, Western blot data (Figures 4e and S3) demonstrated elevated E-Cadherin levels alongside reduced N-Cadherin and Vimentin. The densitometric analysis (Figure 4h) confirmed these observations. Invasion and migration assays (Figure 4g,i) revealed attenuated metastatic capacity, while the corresponding quantitative metrics (Figure 4h,j) verified significant reductions in invasive and migratory behavior. RT-qPCR further indicated elevated E-Cadherin mRNA and diminished N-Cadherin/Vimentin transcripts (Figure 4k–m), highlighting the ability of Zn-Quer NZs to impair both the proliferation and EMT processes in gastric cancer.

### 3.5 Zn-Quer NZs Disrupt Maintenance Levels after ROS Generation by Pharmacological Targeting of NOX4 and Induce Apoptotic Properties in GC Cells

To gain deeper insight into the antitumor mechanism, immunofluorescence and molecular analyses were carried out to evaluate the NOX4 expression and apoptotic activity. Figure 5a–b shows a pronounced decline in NOX4 signals upon Zn-Quer NZ administration, while Annexin V/PI staining (Figure 5c–d) revealed increased early and late apoptotic cell populations. Quantification of these results (Figure 5g–h) underscores the pro-apoptotic effect.

**Figure 5:**
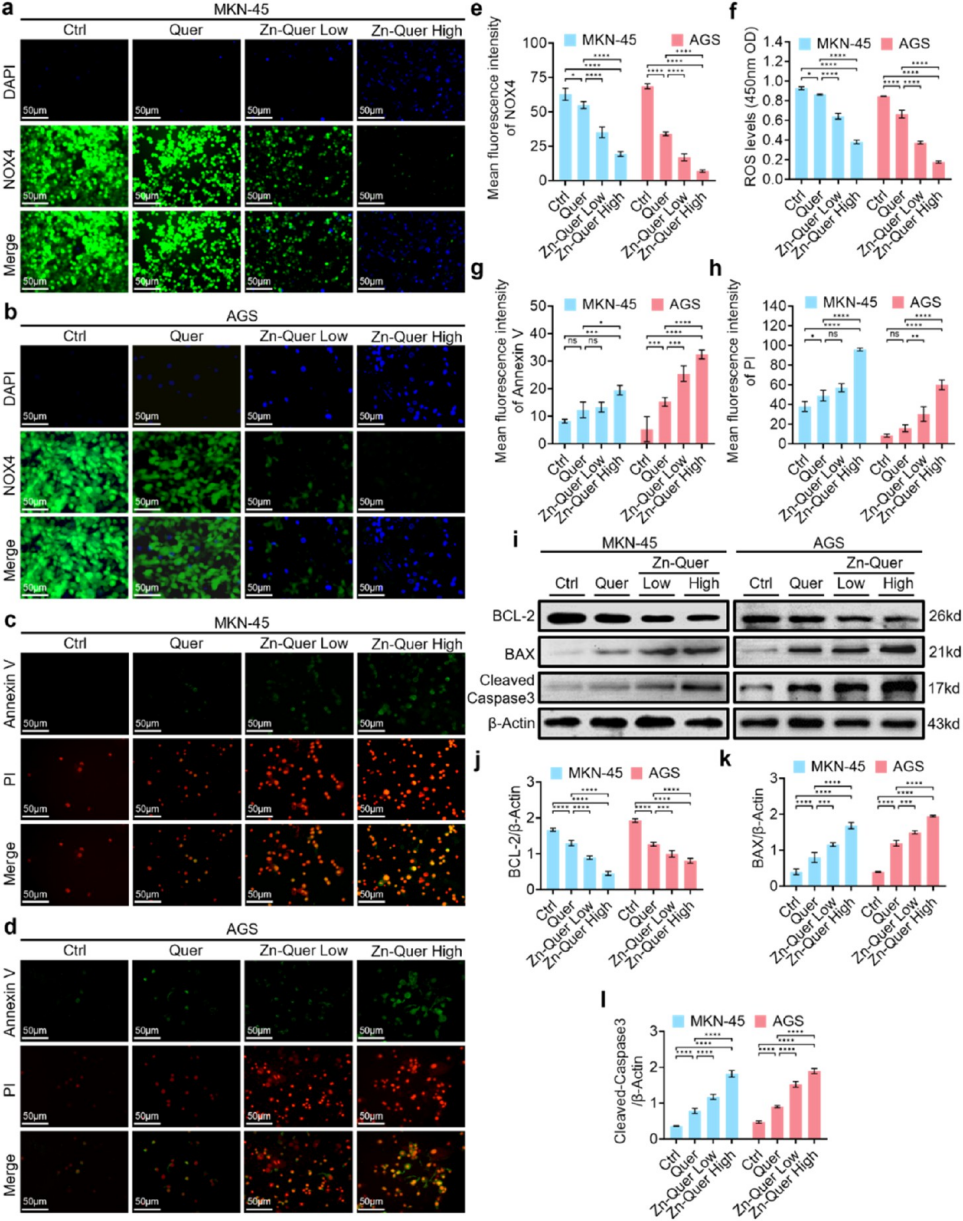
Zn-Quer NZs downregulate NOX4 levels and induce apoptosis in gastric cancer cell. (a, b) Immunofluorescence staining images of NOX4 and DAPI in MKN-45 and AGS cells following varied treatments (Zn-Quer Low = 4 μg/mL, Zn-Quer High = 16 μg/mL). (c, d) Fluorescence images of MKN-45 and AGS cells stained with Annexin V/PI after different treatments­(Zn-Quer Low = 4 μg/mL, Zn-Quer High = 16 μg/mL). (e) Mean fluorescence density derived from the quantified images in sections (a) and (b) (*n* = 3). (f) Fluorescence zymography was used to assess ROS levels in MKN-45 and AGS cells stained with DCFH-DA following various treatments (*n* = 3). (g) Average fluorescence density calculated from the Annexin fluorescence images quantified in panels (c-d) (*n* = 3). (h) Average fluorescence density calculated from the PI fluorescence images quantified in panels (c, d) (*n* = 3). (i) Western blot analysis was used to detect the expressions of BCL-2, BAX, and cleaved-Caspase3. (j–l) Quantification of BCL-2, BAX, and Cleaved-Caspase3 expression in panel (i) was performed (*n* = 3).

Western blot findings (Figure 5i) indicated that Zn-Quer NZs suppressed BCL-2 while upregulating BAX and Cleaved-Caspase3. As illustrated in Figure 5j–l, a dose-dependent decrease in BCL-2 and concomitant increases in BAX and Cleaved-Caspase3 were confirmed. The results of the RT-qPCR quantitative detection of BCL-2 and BAX mRNA are shown in Figure S4, which show the same trend. These data suggest that Zn-Quer NZs trigger apoptosis in gastric cancer cells via NOX4 modulation and shifts in the BCL-2/BAX/Caspase3 balance.

### 3.6 Zn-Quer NZs Reprogramming of Tumor Properties in Xenograft Models

The in vivo antitumor efficacy of Zn-Quer NZs (25 mg/kg) was studied using a xenograft model. Considering the absorption process of nanomedicines through the digestive tract, we first determined the stability of Zn-Quer in the gastric acid microenvironment (Figure S5A). The results showed that Zn-Quer degraded significantly in a simulated gastric environment. However, we measured the enrichment of Zn-Quer and Quer in subcutaneous tumors at different time points (Figure S5B). Zn-Quer showed better enrichment in tumors than Quer, indicating that Zn-Quer has a good drug delivery system. In addition, we detected the content of Quer in lower gastrointestinal tissue, blood, and tumors at different time points (Figure S6), and the results showed that Zn-Quer was effectively enriched in tumors. Figure 6a–b depicts a prominent decrease in tumor volume in treated groups compared to control and Quercetin­(25 mg/kg) groups. Immunofluorescence images in Figure 6c–d demonstrated reduced NOX4, VEGF, and *K*_i_-67 expression, accompanied by alterations in EMT markers (N-Cadherin, E-Cadherin, Vimentin), indicating a multipronged antitumor response.

**Figure 6:**
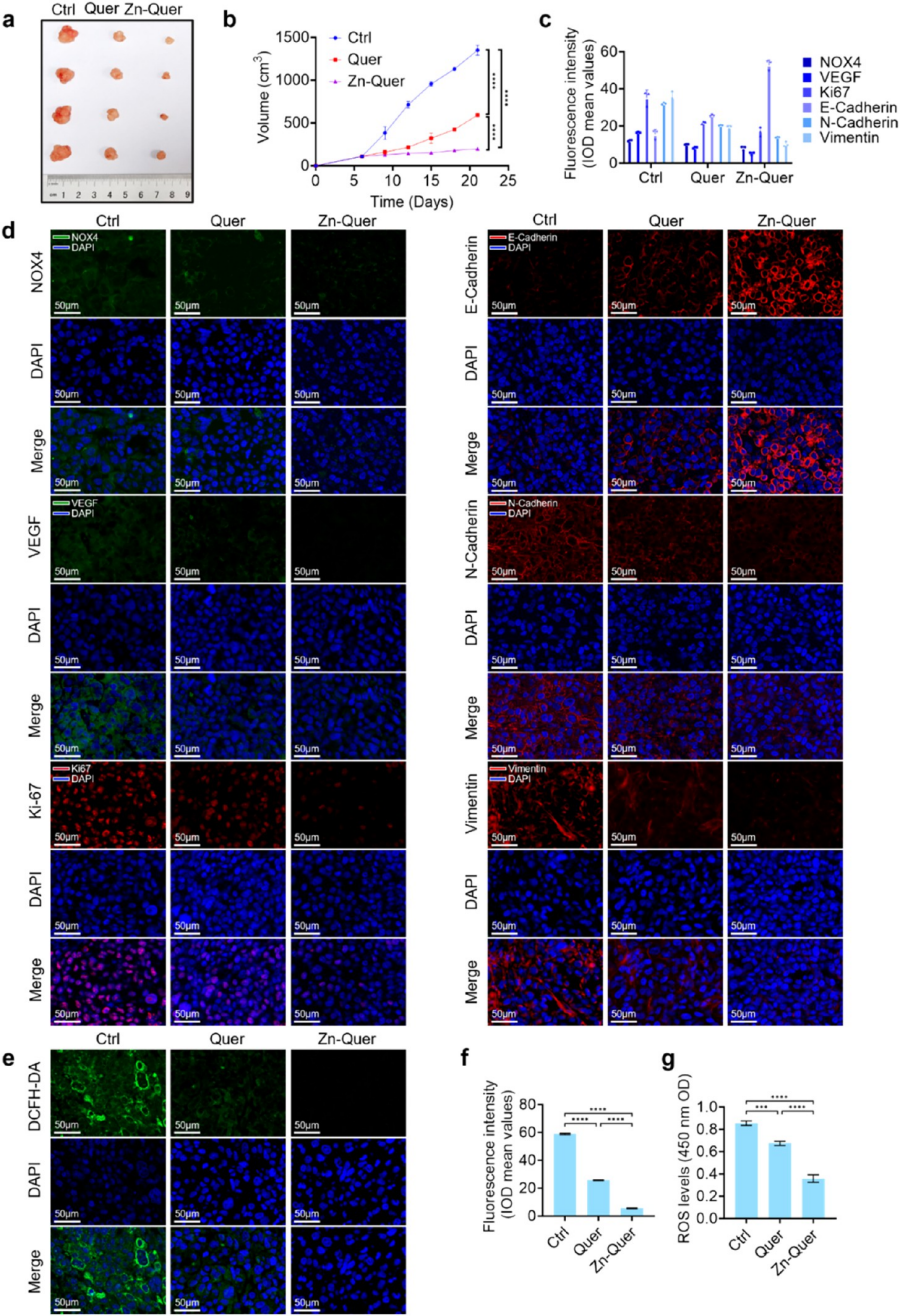
Effect of Zn-Quer NZs on tumor growth in a transplanted tumor model. (a–b) Tumor volume analyses in different treatment groups. (c–d) Immunofluorescence assessment of NOX4, VEGF, *K*_i_-67, and EMT markers (*n* = 3). (e) ROS detection in tumor tissues via DCFH-DA. (f) Quantification of ROS fluorescence intensities in panel (e) (*n* = 3). (g) Fluorescence zymography confirming modulation of ROS (*n* = 3).

A substantial reduction in ROS levels was noted in tumor tissues stained with DCFH-DA (Figure 6e–f), while fluorescence zymography (Figure 6g) corroborated these findings. Taken together, these observations suggest that Zn-Quer NZs restrict tumor growth and modulate critical oncogenic pathways involving oxidative stress, angiogenesis, and EMT.

### 3.7 Biosafety of Zn-Quer NZs In Vivo

A biosafety study was performed in tumor-bearing BALB/c mice treated with Zn-Quer NZs at 20, 40, or 80 mg/kg for 21 days. As depicted in Figure 7a, hematoxylin and eosin (HE) staining revealed no observable damage in the heart, liver, spleen, lung, or kidney. To further evaluate spleen toxicity, we extracted spleen tissue and detected the expression of cytokines IL-6, IL-8, and TNF-α (Figure S7). The results showed that different treatment groups had the same spleen toxicity as the control group, indicating that Zn-Quer has a good biocompatibility. Serum cytokines IL-6, IL-8, and TNF-α remained within normal ranges (Figure 7b–d) and malondialdehyde (MDA; Figure 7e).

**Figure 7:**
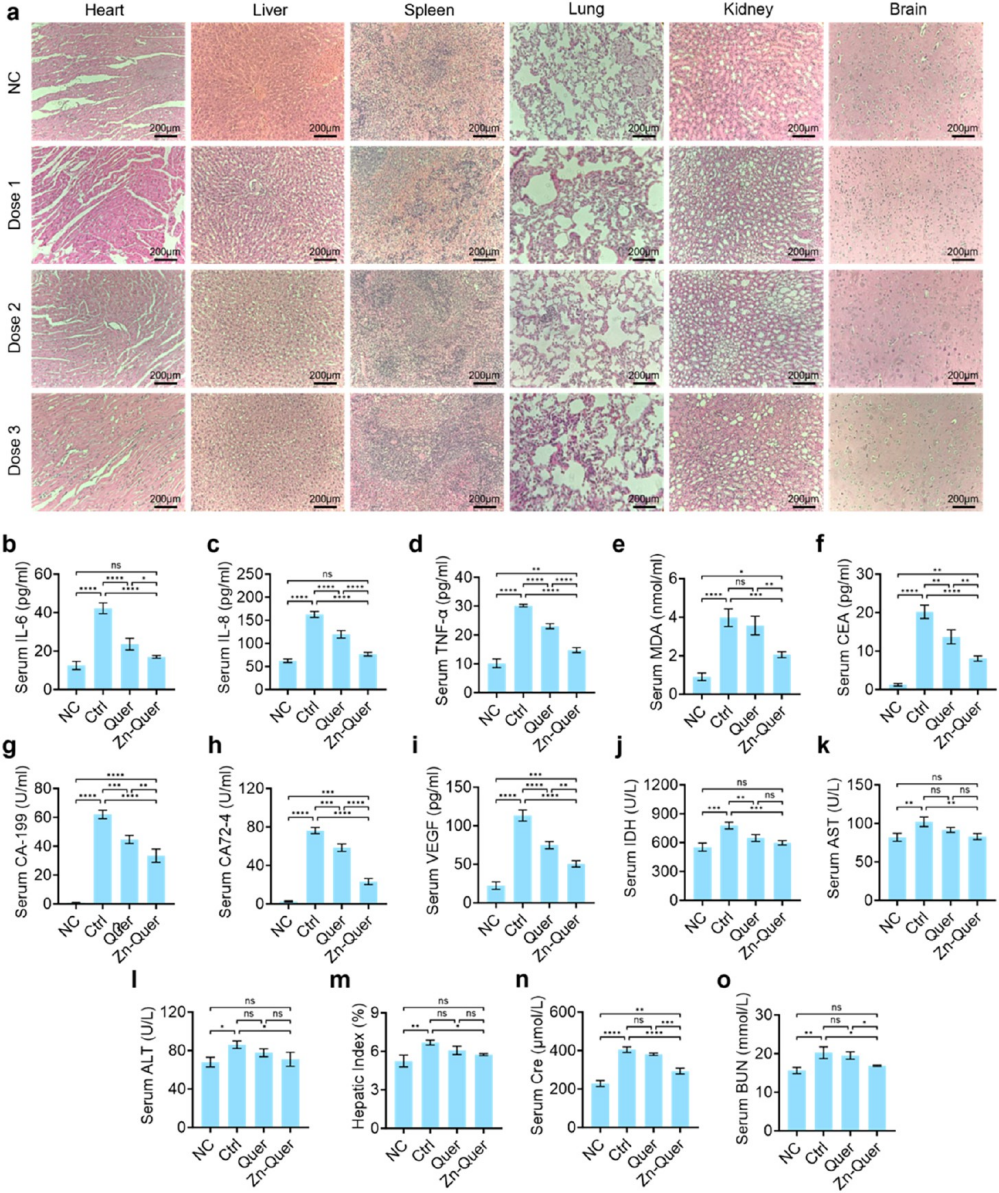
Safety evaluation of Zn-Quer NZs.(a) H&E staining of major organs after 21-day treatment of tumor-bearing BALB/c mice (Dose 1:25 mg/kg Zn-Quer NZs; Dose 2:50 mg/kg Zn-Quer NZs; Dose 3:100 mg/kg). (b–d) Serum cytokine levels for IL-6, IL-8, and TNF-α were measured (*n* = 3). (e) Serum MDA concentrations. (f–h) Tumor biomarkers (CEA, CA-199, CA72–4; *n* = 3).(i) Serum VEGF levels (*n* = 3). (j) LDH levels in serum (*n* = 3). (k–m) Liver function indicators (AST, ALT, Hepatic Index; *n* = 3). (n–o) Kidney function markers (Creatinine, BUN; *n* = 3).

Tumor markers CEA, CA-199, and CA72–4 exhibited pronounced reductions (Figure 7f–h), and serum VEGF also decreased (Figure 7i). In addition, lactate dehydrogenase (LDH) remained stable (Figure 7j), and hepatic and renal function indicators (AST (Aspartate Aminotransferase), ALT (Alanine Aminotransferase), Hepatic Index, Creatinine, and BUN (Blood Urea Nitrogen)) stayed within normal limits (Figure 7k–o). These findings confirm that Zn-Quer NZs possess a favorable safety profile, supporting their use in therapeutic applications.

## 4 Discussion

The present study demonstrates that Zn-Quer NZs offer notable anticancer effects against GC. The nanoscale assemblies were thoroughly characterized by TEM, FTIR, XRD, and XPS, indicating stable crystalline structures and confirming the successful coordination of Zn^2+^ with quercetin. In vitro evaluations revealed potent ROS-scavenging capabilities and enzymomimetic properties, with minimal cytotoxicity toward nonmalignant GES-1 cells. Additional results established that Zn-Quer NZs inhibit GC cell proliferation, suppress angiogenesis and EMT, and downregulate NOX4 while promoting apoptosis through the BCL-2/BAX/Caspase3 pathway. Finally, in vivo xenograft experiments showed reduced tumor growth accompanied by a favorable safety profile, thus emphasizing the translational potential of Zn-Quer NZs.

In vitro experiments have demonstrated that Zn-Quer NZs can effectively scavenge ROS and exhibit enzymomimetic activities, reflecting a capacity to maintain the redox balance in cultured gastric epithelial cells. Assessments of cytotoxicity indicate that these nanoparticles remain biocompatible at clinically relevant doses, thereby preserving healthy tissue function. Investigations into antiproliferative effects further confirm the inhibitory impact of Zn-Quer NZs on GC cell viability, while analyses of epithelial–mesenchymal transition markers reveal that they can counteract crucial steps involved in tumor invasiveness. These findings suggest that beyond simple antioxidant action, Zn-Quer NZs also possess a multifaceted mechanism capable of influencing several oncogenic processes.

Oral administration of Zn-Quer NZs has been associated with reduced tumor growth in vivo models, diminished oxidative stress in tumor tissues, and lower expression levels of pro-angiogenic markers. Histological and biochemical evaluations reveal minimal evidence of adverse effects on major organs, supporting the notion that Zn-Quer NZs may be administered safely. In tandem, tumor biomarkers linked to proliferation, survival, and metastasis appear to be downregulated, underscoring the breadth of antitumor activities.

A principal achievement of this work lies in demonstrating how metal–ligand coordination can substantially enhance the pharmacokinetic and pharmacodynamic properties of quercetin, a naturally derived compound with broad bioactivities yet limited clinical utility due to inadequate solubility and stability. Similar coordination-driven strategies have recently been reported to markedly enhance quercetin stability, loading efficiency, and bioactivity. For instance, Wang et al. developed a metal–quercetin–histidine cocoordinated nanoparticle that displayed strengthened structural stability, superior ROS-scavenging capability, and potent immunomodulatory effects, demonstrating that metal–ligand assembly provides an efficient platform to potentiate flavonoid-based therapeutics.^17^ These findings align with our results and further support that metal–polyphenol coordination is an effective approach to overcome quercetin’s intrinsic limitations in solubility and metabolic instability.

By combining quercetin with zinc in a nanoscale form, this strategy can protect quercetin from rapid metabolism and enhance its long-lasting effect in tissues. Given the elevated NOX4 expression and ROS accumulation in gastric cancer, Zn-Quer NZs enable targeted and controlled drug delivery to tissues, effectively mitigating oxidative-stress-induced cell inflammation and malignant transformation. The integrated targeting of ROS homeostasis, angiogenesis, EMT, and apoptotic pathways signifies a comprehensive therapeutic strategy for gastric cancer management. The comprehensive in vitro and in vivo approaches, utilizing molecular assays, imaging techniques, and xenograft modeling, support the translational significance of these findings and highlight Zn-Quer NZs as a promising candidate for clinical applications.

Recent investigations have underscored the appeal of multifunctional metal–organic frameworks and coordination polymers as drug carriers in oncology due to their high loading capacity, improved solubility, and enhanced tissue penetration.^18–20^ In previous studies, zinc-based nanocomplexes have attracted increasing attention for their synergistic effects in amplifying flavonoid bioactivity and regulating oxidative stress.^21,22^ This study’s Zn-Quer NZs design leverages zinc ions as stabilizing agents to preserve quercetin’s antioxidant and anti-inflammatory properties, aligning with current trends.

Studies have shown that the accumulation of ROS in gastric cancer cells is closely related to tumor proliferation, promotion of invasion, distant metastasis, angiogenesis, and regulation of the tumor microenvironment.^23–27^ Excessive accumulation of ROS can lead to DNA damage, promote mutation events, and alter the expression of tumor-related genes._ROS can also stimulate various MAPK family pathways and receptor tyrosine kinases (RPTKs), facilitating tumor metastasis. ROS help maintain cytoskeletal dynamics, participate in the formation of pseudopodia in tumor cells, and facilitate tumor invasion and metastasis.^28,29^ Furthermore, ROS upregulate the expression of matrix metalloproteinases (MMPs), participate in membrane degradation, and enable primary tumor cells to detach from the extracellular matrix.^30,31^ ROS can facilitate gastric cancer growth and metastasis by enhancing the inflammatory responses and angiogenesis.

Although many nanozyme-based cancer therapies aim to elevate ROS to cytotoxic levels, accumulating evidence indicates that gastric cancer cells actually rely on a moderately elevated yet nonlethal ROS state to maintain malignant phenotypes.^32^ This redox balance activates NOX4-dependent signaling, which in turn drives EMT, enhances proliferative and survival capacity, and suppresses apoptosis. NOX4-generated ROS have been shown to promote EGFR upregulation and anoikis resistance in gastric cancer cells, further supporting its role as a key pro-tumorigenic node.^33^ Likewise, ROS-mediated redox signaling is essential for orchestrating EMT programs, cytoskeletal remodeling, and metastatic dissemination.^34^ Therefore, reducing ROS (particularly NOX4-derived ROS) does not simply protect normal cells but directly disrupts the oxidative signaling machinery required for tumor aggressiveness. In our study, Zn-Quer NZs decreased intracellular ROS to a nononcogenic range, resulting in NOX4 suppression, EMT reversal, inhibition of pro-survival BCL-2 signaling, and resensitization to apoptosis. These findings indicate that ROS scavenging in this context does not counteract anticancer activity; instead, it selectively targets the redox addiction that sustains gastric cancer progression.

Reactive oxygen species levels play a complex role in the behavior of cancer cells. Elevated ROS levels can induce apoptosis and damage cellular components, inhibiting tumor growth and making them a focus of anticancer research. This oxidative stress induction is integral to the therapeutic mechanisms of chemotherapy and radiotherapy. However, high ROS levels are often a hallmark of cancer cells, promoting the development and maintenance of cancer by enhancing the cell proliferation and survival. Tumor cells have an enhanced ability to tolerate ROS and metastasize under mild oxidative stress conditions.^35^ Oxidative stress, driven by ROS-induced DNA damage, mutations, and oncogenic signaling, contributes to cancer progression by compromising genomic integrity, inducing gene mutations, activating oncogenes, inactivating tumor suppressor genes, and promoting genomic instability and chronic inflammation.^36–38^ Inhibiting the ROS generation pathway or using antioxidants to enhance the clearance of ROS can protect normal cells from malignant transformation and inhibit the early stages of tumorigenesis by preventing or reducing intracellular ROS.^39,40^ While ROS scavenging offers a promising anticancer approach, its efficacy can differ based on the unique molecular characteristics of cancer cells and their capacity to adapt to oxidative stress changes.^41^ Recent work has emphasized that the therapeutic manipulation of ROS is not limited to simple elimination but increasingly involves precise catalytic modulation. Zhong et al. demonstrated that nanozymes with dynamic metal–ligand coordination can couple catalytic ROS regulation with postcatalytic ion release, enabling more controlled redox modulation within the tumor microenvironment. This paradigm highlights that nanozyme-based ROS regulation can be both catalytic and adaptive, supporting the rationale for our Zn-Quer NZs in targeting NOX4-driven oxidative signaling in gastric cancer.^42^ ROS signaling is essential for EMT to promote tumor progression. The NOX family, a key source of ROS mainly found in the cytoplasm, is linked to EMT promotion, invasion, angiogenesis, drug resistance, and other cancer cell phenotypes.^43,44^ Studies on ROS from mitochondrial circuits focus on mediating apoptosis; however, lack of selectivity may lead to extensive cell damage and side effects, and the complexity of the tumor microenvironment leads to uncertainty in therapeutic effects, with inherent risks of promoting tumor cell metastasis.^45^ This suggests the potential of strategies based on inhibiting NOX4 and regulating ROS in blocking malignant transformation and reducing the malignant behavior of cancer cells.

Angiogenesis is one of the key processes of tumor growth and metastasis, and ROS has been shown to promote angiogenesis, especially in the tumor microenvironment. As an important ROS-producing enzyme, the expression of NOX4 in gastric cancer cells is closely related to ROS levels. Our findings demonstrated that Zn-Quer NZs effectively reduced NOX4 expression and ROS production in gastric cancer cells while also downregulating HIF-1α to decrease vascular endothelial growth factor expression. NOX4 has a direct effect on the level of intracellular ROS and also affects tumor cell proliferation, survival, and angiogenesis Du et al. The study demonstrated that depleting NOX4 markedly suppresses invasion, proliferation, EMT, and MMP7 expression in gastric cancer cells, thereby hindering gastric cancer progression in vivo.^46^ Knockdown of NOX4 reduces ROS secretion and EGFR expression, promotes anoikis apoptosis of gastric cancer cells, and inhibits distant metastasis of GC.^33^ Tang et al. enhanced the apoptosis of gastric cancer cells by blocking the activation of the NOX4-derived ROS and Gli1 signaling.^47^

The experimental scope primarily addresses ROS-related pathways but does not fully elucidate how Zn-Quer NZs might interact with the immune landscape of the tumor microenvironment, which is highly relevant for advanced therapeutic interventions. The biodistribution analysis, although positive, could be expanded to include prolonged treatment regimens that test accumulation and clearance patterns over extended timelines. The feasibility of large-scale manufacturing, including cost-effectiveness and reproducibility, remains to be explored. Investigations focusing on possible synergy with established treatments—such as immunotherapies or chemotherapeutics—would further define the broader clinical utility of nanozymes. These directions may yield a more comprehensive understanding of Zn-Quer NZs and help refine their design for more targeted and personalized gastric cancer interventions.

## 5 Conclusions

In conclusion, our study demonstrated that Zn-Quer NZs could effectively reprogram the deterioration characteristics of gastric cancer cells by dynamically modulating the level of ROS in multiple phases, showing powerful inhibition of EMT, promotion of apoptosis, and reduction of angiogenesis in gastric cancer cells, and in vitro and in vivo experiments demonstrated the great potential of Zn-Quer NZs as a novel targeting of ROS coregulation for the prevention and treatment of gastric cancer. The in vitro and in vivo experiments showed that Zn-Quer NZs, as a novel target of ROS coregulation in gastric cancer, have great potential to be applied in other ROS-related diseases and other tumor systems.

## Supplementary Material



## Data Availability

The data that support the findings of this study are available from the corresponding authors upon reasonable request.

## Notes

Sequences of primers (Table S1); the analysis of Zn-Quer for particle size (A) and potential (B) (Figure S1); evaluation of drug combination efficacy. (A) Curves showing the effect of drug dosage. (B) Zn-Quer drug combination index (Figure S2); quantification of N-Cadherin, E-Cadherin, and Vimentin expression (Figure 4e) (*n* = 3) (Figure S3); relative mRNA expression of BCL-2, BAX (*n* = 3) (Figure S4); Zn-Quer drug delivery assessment. (A) Zn-Quer drug release in a simulated gastric acid environment. (B) Accumulation of Zn-Quer and Quer at the tumor site at different time points (Figure S5); Zn-Quer content changes over time in different tissues (Figure S6) (PDF)
