# Supplementary material for: Zn-Quer Nanozymes Reprogram the Malignant Phenotypic Transformation of Gastric Cancer Cells via Cascade Reactive Oxygen Species Coordination
Source: ACS Appl Mater Interfaces. 2026 Jan 8;18(2):3499–514. doi: 10.1021/acsami.5c17346 (PMC12828714; doi:10.1021/acsami.5c17346)
Supplement: Supplementary file 1 [file am5c17346_si_001.pdf]

Supporting Information

Zn-Quer Nanozymes Reprogram the Malignant Phenotypic Transformation of Gastric Cancer Cells via Cascade Reactive Oxygen Species Coordination

Heng Jiang[a,1], Jiahao Wang[a,1], Siyu Gui[b,1], Sensen Niu[c,1], Guangzheng Lin[d], Hui Yuan[a], Yongqi Wu[a], Chuhan Zhou[a], Jingjing Tang, Qiao Mei[d]*, Lianbang Zhou[a]*

[a]Department of General Surgery, the Second Affiliated Hospitalof Anhui Medical University, Hefei 230601, China.

[b]Department of Urology, the Second Affiliated Hospitalof Anhui Medical University, Hefei 230601, China.

[c]Department of Gastroenterology, the First Affiliated Hospitalof Anhui Medical University, Hefei 230022, China.

[d]Department of Ophthalmology, Shanghai General Hospital, Shanghai Jiao Tong University School of Medicine, Shanghai 200025, China.

[1]Heng Jiang, Jiahao Wang, Siyu Gui, and Sensen Niu are equal contributors to this work.

[*]Corresponding authors: Lianbang Zhou, zlbahmu@163.com; Qiao Mei, meiqiao@hotmail.com

Primers	Sequences
NOX4-F	TGGGGCTAGGATTGTGTCTAAGCAG
NOX4-R	CGGCACATGGGTAAAAGGATAAGGC
N-Cadherin-F	TCCGACGAATGGATGAAAGACCCAT
N-Cadherin-R	CAGGGAGTCATATGGTGGAGCTGTG
E-Cadherin-F	ACTGACACCAACGATAATCCTCCGA
E-Cadherin-R	CAGCATCAGTCACTTTCAGTGTGGT
Vimentin-F	TGGAAGAGAACTTTGCCGTTGAAGC
Vimentin-R	AGCAGGTCTTGGTATTCACGAAGGT
BAX-F	TAACATGGAGCTGCAGAGG
BAX-R	CAGTTGAAGTTGCCGTCAG
BCL-2-F	ATTAAGGGACAGACAGGAGC
BCL-2-R	TAAGCTGGAATCCTGAGCA
VEGF-F	GAGTACCCTGATGAGATCGAGTAC
VEGF-R	CATAATCTGCATGGTGATGTTGGAC

Table S1. Sequences of primers.

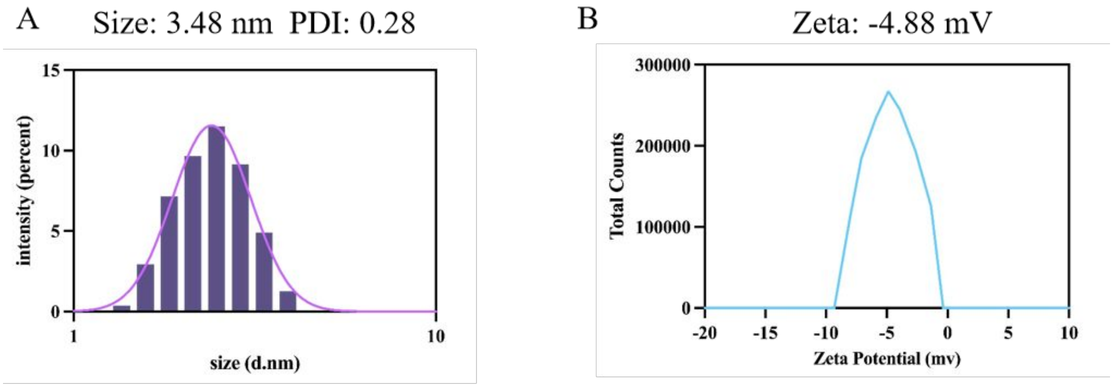

Fig. S1. The analysis of Zn-Quer for particle size (A) and potential (B)

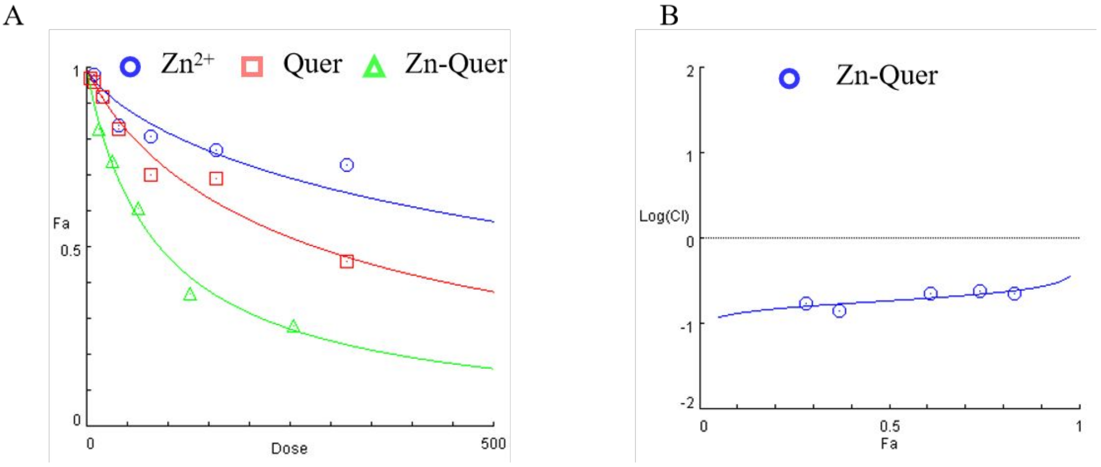

Fig. S2. Evaluation of drug combination efficacy. (A) Curves showing the effect of drug dosage. (B) Zn-Quer drug combination index.

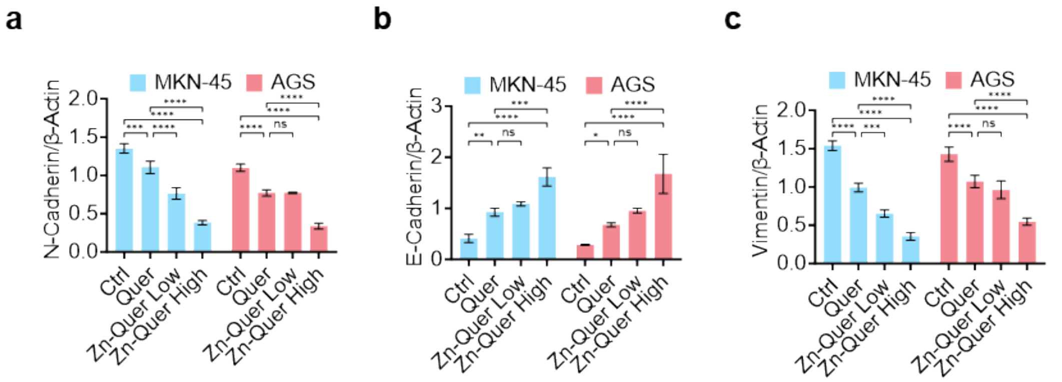

Figure S3. Quantification of N-Cadherin, E-Cadherin, and Vimentin expression in (Fig. 4e) (n=3).

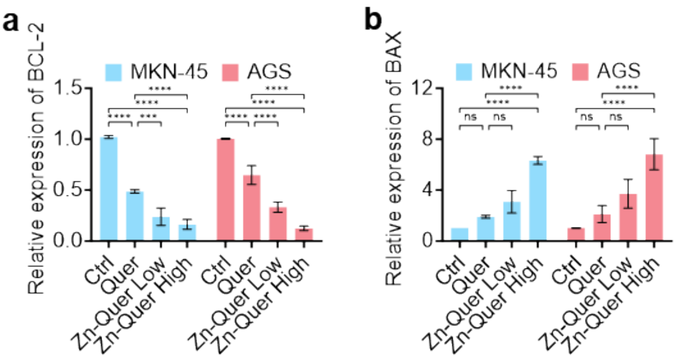

Figure S4. Relative mRNA expression of BCL-2, BAX (n=3).

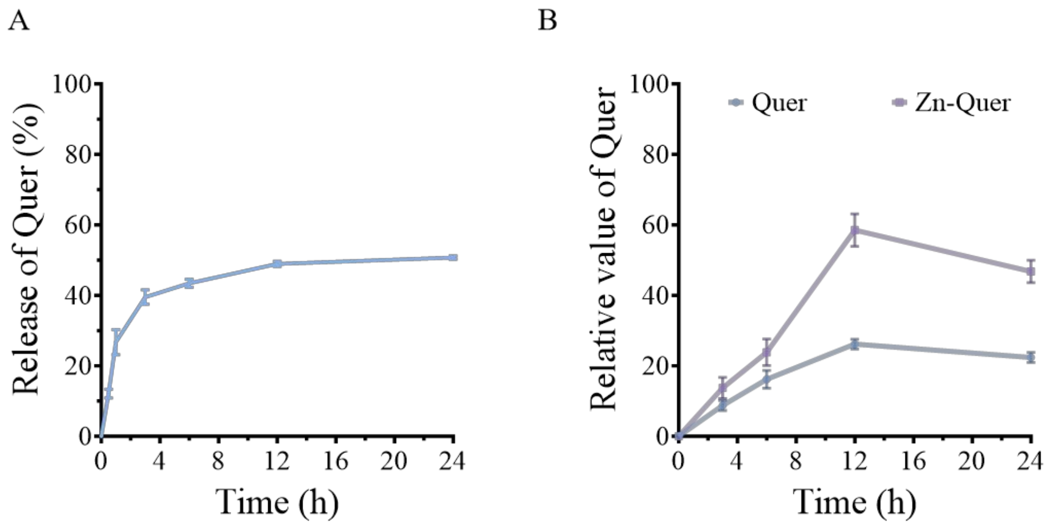

Fig. S5. Zn-Quer drug delivery assessment. (A) Zn-Quer drug release in a simulated gastric acid environment. (B) Accumulation of Zn-Quer and Quer at the tumor site at different time points.

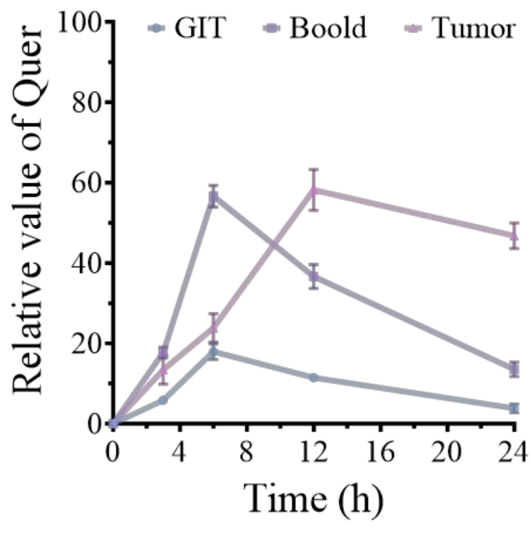

Fig. S6. Zn-Quer content changes over time in different tissues.

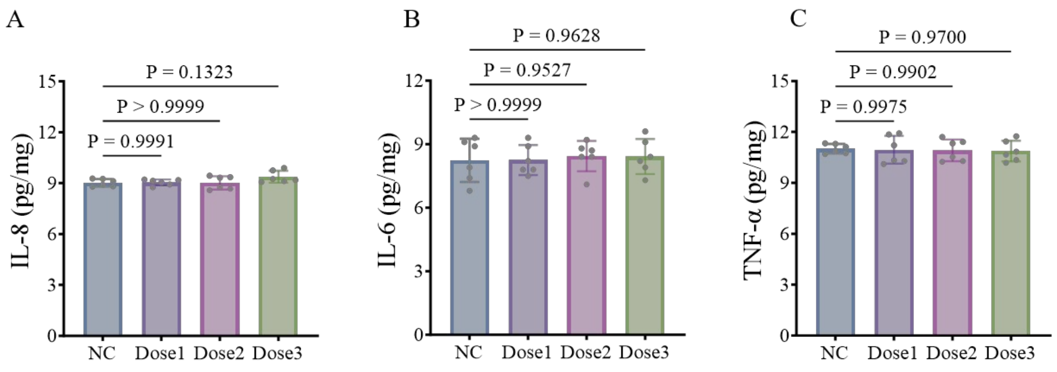

Fig. S7. Zn-Quer for assessing cytotoxicity of spleen cells. Detection of cytokines IL-8 (A), IL-6 (B), and TNF-α (C) in spleen tissue.
